# Rural community engagement and cultural considerations in the design and deployment of remote health monitoring systems: A scoping review

**DOI:** 10.1111/jrh.70141

**Published:** 2026-04-07

**Authors:** Kelly Reed, Victoria M. Petermann, Rhys D. Nicholas, To‐Trinh (Trish) V. Truong, Virginia LeBaron

**Affiliations:** ^1^ School of Nursing University of Virginia Charlottesville Virginia USA

**Keywords:** remote heath monitoring systems, rural health, scoping review, stakeholder engagement

## Abstract

**Purpose:**

Remote health monitoring systems (RHMS) use technology (e.g., wearables, smartphones) to track health‐related information outside of traditional health care settings and hold great promise to enhance access to care for rural residents. To be most effective, RHMS must meet the unique needs and preferences of rural residents. Therefore, the aim of this scoping review is to explore if, and how, RHMS in the United States (US) are designed and deployed with consideration for the cultural and contextual needs of rural residents.

**Methods:**

PubMed, CINAHL, and Web of Science were searched using a combination of keyword and MeSH terms related to rural and remote health technology. Included articles were original research studies published between 2014 and 2024 that focused on the design and/or deployment of RHMS for adults in the US and included rural participants.

**Findings:**

An initial 9175 studies were identified and screened, with 61 unique studies selected for analysis. Of these studies, 50.8% (*n* = 31) provided a clear, operational definition of “rural.” Stakeholder engagement in the design/deployment of RHMS was described in 63.9% (*n* = 39) of studies; however, few studies engaged caregivers (9.8%, *n* = 6). Approximately 44.3% (*n* = 27) of studies explicitly modified RHMS for low broadband access.

**Conclusions:**

Three key themes emerged: limited stakeholder engagement, ongoing infrastructure barriers, and significant variability in how the cultural and contextual considerations of rural residents were incorporated into RHMS design and deployment. As RHMS are increasingly integrated into health care, it is critical to advance approaches that meaningfully support the health and well‐being of rural residents.

## Graphical abstract

This scoping review examines how remote health monitoring systems (RHMS) in the United States incorporate the cultural and contextual needs and preferences of rural residents. Across 61 studies, definitions of “rural” vary, and stakeholder engagement is inconsistent, with limited caregiver involvement. Infrastructure barriers, particularly broadband access, are not consistently addressed. Overall, findings highlight gaps in the integration of rural‐specific considerations and underscore the need for more inclusive, contextually tailored RHMS design and deployment.

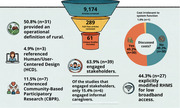

## INTRODUCTION

Approximately 80% of rural residents in the United States (US) are medically underserved.^1^, ^2^, ^3^ Health care provider shortages, long travel distances for care, unreliable transportation, high rates of poverty, low rates of health insurance, and low digital and health literacy can all contribute to poorer health outcomes and shorter life expectancies for rural residents.^1^, ^2^, ^3^, ^4^, ^5^ Digital health technologies, including telemedicine,^6^ telehealth,^7^ and remote health monitoring systems (RHMS),^8^, ^9^ have great potential to address barriers to care and improve health outcomes for rural residents. RHMS use a range of technologies to track health‐related information outside of the traditional health care setting^10^, ^11^, ^12^, ^13^ and may include vital sign devices,^11^, ^14^ blood glucose meters,^11^, ^15^ environmental sensors,^16^ wearables,^11^, ^17^ and applications on smartphones and smartwatches.^11^, ^18^ RHMS represent a form of remote patient monitoring; however, RHMS is the preferred, broader term for this review, as these systems can also monitor caregivers or track overall wellness in healthy (nonpatient) individuals. RHMS are increasingly being used to monitor and improve outcomes for a wide variety of health conditions, including hypertension management, pain management for advanced cancer patients, and rehabilitation after discharge from the hospital.^8^, ^11^, ^19^, ^20^

Specific to the rural context, several recent studies and reviews have confirmed that RHMS can facilitate timely interventions and more personalized care for a wide variety of health conditions while reducing barriers related to distance and limited local resources.^21^, ^22^, ^23^, ^24^, ^25^ Leading rural organizations, including the National Rural Health Association (NRHA), endorse that RHMS hold great promise to benefit rural populations in the US.^26^, ^27^ This potential is also reflected in national policy; the Rural Patient Monitoring Access Act, which explicitly acknowledges the value of RHMS for improving rural health outcomes, is currently under consideration.^28^

RHMS implementation in rural contexts can be hindered by a combination of structural and cultural barriers, including limited broadband access,^29^, ^30^, ^31^, ^32^ high costs,^8^, ^33^, ^34^ low digital literacy,^31^, ^35^, ^36^ and community‐level concerns, such as distrust in the health care system^37^ and heightened sensitivity to privacy and data security.^38^, ^39^, ^40^ The NRHA and US Congress acknowledge that these structural and cultural barriers must be addressed to ensure equitable access to RHMS.^34^, ^39^ Therefore, to be optimally effective, RHMS must be designed and deployed with consideration of the unique needs and preferences of rural end‐users: rural residents, their caregivers, and health care teams.^4^, ^8^, ^19^, ^21^, ^22^, ^23^, ^24^, ^29^, ^30^, ^31^, ^33^, ^34^, ^35^, ^37^, ^38^, ^40^, ^41^ Engaging these stakeholders can help address local technology preferences and capacities,^38^, ^39^, ^40^ affordability and accessibility,^8^, ^29^, ^30^, ^31^, ^33^, ^34^, ^38^, ^40^, ^42^ digital literacy gaps,^31^, ^35^, ^36^ privacy concerns,^38^, ^39^, ^40^ and trust in the health care system,^37^, ^38^, ^40^ while also improving usability,^22^, ^43^ integration into clinical workflows,^8^, ^18^, ^22^, ^37^ and the likelihood of sustained adoption.^30^, ^39^, ^40^, ^41^ Similarly, the Agency for Health care Research and Quality emphasizes that engaging with rural patients is essential for tailoring RHMS to address broadband limitations, device access, and linguistic or cultural differences, thereby ensuring more equitable and “person‐centered” care.^11^

Despite this recognition, no existing review has, to our knowledge, systematically assessed the extent to which RHMS have been designed and deployed with meaningful rural stakeholder engagement or considerations for the unique context of rural areas. This study fills that critical gap by presenting an overview of how rural end‐users are—or are not—engaged in RHMS design and deployment, contributing to broader efforts to tailor health technologies to diverse contexts and communities.^44^ Therefore, the purpose of this scoping review is to explore if, and how, RHMS in the US were designed and deployed with consideration for the cultural and contextual needs of rural end‐users. Without intentional engagement of rural residents, caregivers, and health care teams, we argue that RHMS risk limited adoption, poor accessibility and usability, and missed opportunities to reduce rural health disparities.^4^, ^8^, ^19^, ^21^, ^22^, ^23^, ^24^, ^29^, ^30^, ^31^, ^33^, ^34^, ^35^, ^37^, ^38^, ^40^, ^41^, ^45^ By identifying if, and to what extent, rural end‐users are involved in RHMS design and deployment, this review offers critical insights for researchers, engineers/technical developers, and policymakers who seek to ensure optimally effective digital health interventions. The findings can highlight gaps in relevance and responsiveness of RHMS for rural‐end users, inform best practices for participatory design, and support policy efforts that prioritize access to RHMS.

## METHODS

### Overview of study design

To comprehensively understand how RHMS were designed and deployed to meet the cultural and contextual needs of rural end‐users, we conducted a scoping review due to the emergent and heterogeneous nature of research on designing and deploying RHMS for rural residents.^46^ Scoping reviews are well‐suited to exploring complex, interdisciplinary topics, mapping key concepts, and identifying knowledge gaps—especially when the evidence base is diverse.^47^, ^48^ To ensure methodological rigor, we followed Arksey and O'Malley's established framework for conducting scoping reviews, which provides a systematic and transparent process for identifying, charting, and synthesizing relevant literature.^44^

### Search strategy

In consultation with a health sciences librarian, we developed the search strategy to identify articles studying RHMS design or deployment with rural end‐users. On June 2024, we searched PubMed, CINAHL Complete (accessed through EBSCO), and Web of Science (Core Collection) for research articles published between 2014 and 2024 using a combination of keywords and MeSH terms related to RHMS and rural (see Table 1 for example PubMed search). For the full search strategy across all databases, (see Supporting Information Data File 1). We exported the results into a Zotero library and then uploaded into Covidence,^49^ a literature review management tool, for further evaluation and screening. Additionally, we searched the grey literature for relevant online sources not found in PubMed, CINAHL, and Web of Science, including federal and state government websites, news articles, and other reports.

**TABLE 1:** Example PubMed search strategy used in scoping review to explore remote health monitoring systems (RHMS) designed and deployed for rural end‐users.

For PubMed, the search strategy used was:
((“telemonitor*”[Title/Abstract] OR “telehome*”[Title/Abstract] OR (“monitor*”[Title/Abstract] AND (“remote”[Title/Abstract] OR “digital*”[Title/Abstract] OR “technolog*”[Title/Abstract] OR “telemedicine”[Title/Abstract] OR “telehealth”[Title/Abstract] OR “physiolog*”[Title/Abstract]))) AND (“rural”[Title/Abstract] OR “rurality”[Title/Abstract] OR “rural population”[MeSH Terms] OR “rural health”[MeSH Terms]) AND (english[Filter]). For CINAHL and Web of Science, the search terms used were: ((telemonitor* OR telehome*) OR (monitor AND (remote OR digital* OR technolog* OR telemedicine OR telehealth OR physiolog*))) AND (rural OR rurality OR “rural population” OR “rural health”)

### Inclusion and exclusion criteria

We included studies published in English in peer‐reviewed journals between 2014 and 2024 that discussed the design (creation and planning) and/or deployment (testing and use with end‐users) of RHMS. On the basis of discussions among our team, and informed by the literature, we defined RHMS as “any individual or combination of electronic devices used to monitor or track health‐related data or outcomes outside of the traditional health care setting.”^8^, ^9^, ^10^, ^11^, ^12^, ^14^, ^15^, ^16^, ^17^, ^18^, ^19^ Examples of RHMS could include vital sign devices,^11^, ^14^ blood glucose meters,^11^, ^15^ environmental/ambient sensors,^16^ wearables,^11^, ^17^ and applications on smartphones and smartwatches.^11^, ^18^ The RHMS could collect data passively (no user engagement required)^11^, ^50^, ^51^ and/or actively (user engagement required).^11^, ^51^ The data collected by the RHMS could be shared with a health care or research team,^11^, ^52^, ^53^ informal caregivers,^54^ or used for self‐monitoring by the individual.^55^, ^56^ Studies were eligible for inclusion if they involved any rural end‐users, though they did not need to target rural populations exclusively. We did not apply a single, uniform definition of “rural” during the screening process. Instead, we evaluated each article to assess how the authors defined “rural.”

In consultation with the literature, the team agreed upon working definitions that distinguished RHMS from other forms of digital health technology, including telemedicine and telehealth.^10^, ^12^, ^53^ For the purposes of this review, telemedicine was considered scheduled, synchronous virtual visits between patients and health care providers,^10^ and telehealth as the broader umbrella term encompassing telemedicine as well as other remote health services, such as online portals.^10^, ^12^ Although RHMS may be considered a type of telehealth or telemedicine, not all telehealth or telemedicine services utilize RHMS. Accordingly, studies of telehealth or telemedicine were included only if an RHMS component was incorporated.

Studies were excluded if they were (1) secondary sources, such as literature reviews; (2) papers whose sole focus was on developing specific technical components of the RHMS (e.g., electrode or sensor development and testing); or (3) discussed RHMS in aggregate with other digital tools but without specific reference to the RHMS design or deployment. Our final article count reflects the number of unique studies; we noted study protocols and articles reporting findings from the same study alongside the primary corresponding article in the data extraction spreadsheet but did not count them as separate studies.

Initial title and abstract screening and full‐text review were conducted by three independent reviewers (K.R., V.P., and T.T.) using Covidence. Discrepancies were addressed through discussion with the research team to reach consensus.

### Full text data extraction

Data elements for full text data extraction were informed by the existing literature^11^, ^41^, ^57^, ^58^, ^59^, ^60^ aims of the scoping review, and team discussion and consensus. Table 2 summarizes the data elements extracted from each included article with examples. See Supporting Information Data File 2 for the complete data extraction tool, which includes all data extraction categories. Data extraction included both quantitative and qualitative elements. Quantifiable study characteristics (those we could count and assign more easily to categories), such as health care focus (e.g., diabetes), RHMS device components (e.g., wearables, smart phones), or data extraction categories with clear yes/no questions (e.g., did they discuss financial considerations of RHMS?), were tallied and summarized descriptively using an Excel file. More qualitatively oriented study characteristics, such as barriers and facilitators to the use of RHMS for rural residents, were compiled in a word document and analyzed across studies to look for patterns. We then reviewed our quantitative and qualitative findings to determine key themes that are described in the Results section.

**TABLE 2:** Extracted study characteristics and remote health monitoring systems (RHMS) features with examples of potential responses.

Study design^[Link]^, ^[Link]^	Primary RHMS phase^a^	Health care focus^a^	What is, or is intended to be, measured/monitored?^a^	Who or what is/is intended to be measured/monitored?^a^	RHMS device components^a^	Where does/will the data go?^a^
Quantitative Qualitative Mixed methods Multiple methods	Planning/Design Deployment/testing	Wellness/Prevention Diabetes Hypertension Cancer Obesity Mental health	Blood pressure Sleep Blood glucose Activity Symptoms (specify) Weight Medication adherence	Individual only Individual + other (e.g., Caregiver) Home	Watch wearable Smartphone Tablet Ambient/Environmental sensors Glucometer Wearable vital signs devices	Self‐monitoring individual Self‐monitoring individual + caregiver Health care provider^c^ Allied health professional^d^ Research team Hospital/Clinic

**Geographic location of target end‐user? Is rural defined using operational definition?**	**At what geographic level is rural defined?** ^[Link]^, ^[Link]^	**What definition is used to classify rural?** ^a^	**Was stakeholder input obtained? (associated DOI)**	**If yes, when was stakeholder input obtained?** ^a^	**What type of stakeholder input was provided?** ^a^	**Do they reference any of the following frameworks/theories?** ^a^
Write‐in Yes No Unclear	County Census tract Zip code Regional (e.g., Appalachia)	Rural‐urban commuting areas FORHP definition (HRSA) Core‐based statistical areas (OMB) CDC rural classification scheme	Yes No Write‐in	Design Deployment Unclear	Patients Rural? Informal Caregivers Rural? Health care Clinicians Rural? Community Members Rural?	Human‐centered design User‐centered design Design thinking Community‐based participatory research No/Unclear

**Did they consider or adapt for broadband access?**	**What broadband considerations or adaptations were made?** ^b^ **Did they exclude participants who did not have broadband?**	**Did they discuss financial considerations of RHMS (e.g., insurance coverage)? If yes, what did they discuss and/or do?**	**Did they adapt for other rural cultural/Contextual considerations?** ^a^	**Lessons learned: barriers to RHMS identified** ^b^	**Lesson learned: facilitators to RHMS identified** ^b^	**Relevant study limitations and recommended next steps (as identified by author)**
Discussed/Adaptions made Discussed but no adaptions made Not discussed/Adapted Design phase: discussed Design phase: not discussed Not necessary	Write‐in Yes No All participants had broadband Design phase; not yet relevant	Yes No Unnecessary for system operation Write‐in	Distance to care Community distrust in health care Privacy/Security concerns Digital literacy No/Unclear Other (describe)	Write‐in	Write‐in	Write‐in

Abbreviation: HRSA, Health Resources and Services Administration.

^a^Select all that apply.

^b^Per study author description.

^c^Health care provider: An individual licensed to deliver health care services, such as diagnosis, treatment, and preventive care, including physicians, physician assistants, nurse practitioners, and nurses.^156^

^d^ Allied health professional: diverse group of non‐physician, non‐nurse health care workers who promote health and wellness, and support health care systems across various settings.^157^

## RESULTS

**FIGURE 1:**
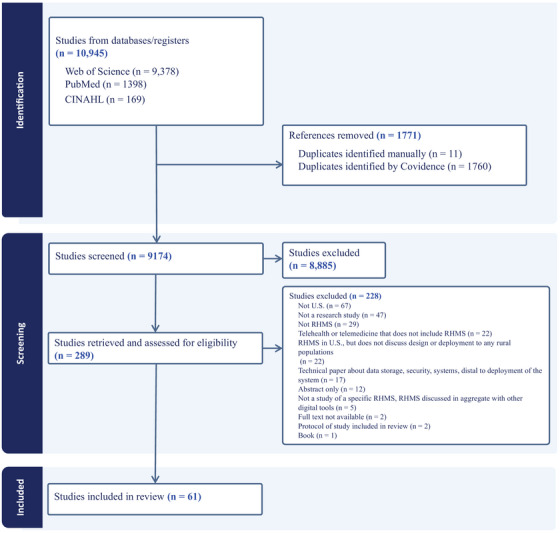
PRISMA diagram illustrating the identification and selection of studies examining the design or deployment of remote health monitoring systems (RHMS) for rural end‐users.

The article selection process is summarized in Figure 1. After the removal of 1770 duplicates, a total of 9175 records remained for title and abstract screening. Of these, 289 articles were selected for full‐text review. Following full‐text assessment, 228 studies were excluded on the basis of eligibility criteria. We did not identify additional relevant articles for data extraction after searching the grey literature. Ultimately, 61 studies were included in the final analysis and are summarized in Tables 3, 4, 5, 6.

**TABLE 3:** Summary of key study characteristics in remote health monitoring systems (RHMS) designed and/or deployed for rural participants, 2014–2016 (*n* = 10).

References	Primary study design*	Primary RHMS phase	Who is being monitored?	Location of end‐user*	Health care focus	What is being monitored?	RHMS device components	Where does the data go?
Giger^98^	Qualitative	Deployment	Individual	Frontier, older adults	Wellness/Prevention, diabetes, other chronic illness	Physiologic, weight	Tablet, wearable vital signs device, digital scale	Health care provider
Petitte^109^	Quantitative	Deployment	Individual	Appalachian population in West Virginia	Cancer	Physiologic, symptoms, weight	Wearable vital signs device	Research team
Riley^112^	Quantitative	Deployment	Individual	Predominantly rural	Heart failure	Physiologic, weight	Smartphone, wearable vital signs device	Allied health professional, community hospital/clinic
Shane‐McWhorter^114^	Quantitative	Deployment	Individual	Rural primary care clinics in Utah	Diabetes, cancer	Physiologic, blood glucose, weight	Wearable vital signs device, digital scale	Health care provider, allied health professional
Siminerio^115^	Qualitative	Deployment	Individual	Rural Western Pennsylvania	Diabetes	Blood glucose	Glucometer	Health care provider, rural hospital/clinic, academic hospital/clinic
Cherry^65^	Qualitative	Deployment	Individual	Rural veterans in underserved locations	Stroke	Mobility/Activity	Other wearable, processing unit with pneumatic pump	Health care provider
Chen^64^	Mixed method	Design	Individual and home/external entity	Rural mountainous Appalachia	Cancer	Physiologic, mobility/Activity, nutrition	In‐home telemonitoring system	Self‐monitoring individual, allied health professional, research team
Foley^105^	Quantitative	Deployment	Individual	A seven‐county service area in central North Carolina	Obesity	Mobility/Activity, weight, nutrition	Other wearable	Self‐monitoring individual, health care provider, research team, community hospital/clinic
Frisbee^106^	Qualitative	Design	Individual and caregiver	Unclear	Serious physical or mental injury	Physiologic, mobility/activity, symptoms, nutrition, medication adherence	Tablet	Self‐monitoring individual, caregiver, research team
Peng^96^	Multi‐method	Design	Individual	Midwest region of the US	Diabetes	Blood glucose, mobility/activity	Smartphone	Self‐monitoring individual

* Per study author description.

**TABLE 4:** Summary of key study characteristics in remote health monitoring systems (RHMS) designed and/or deployed for rural participants, 2017–2019 (*n* = 12).

References	Primary study design*	Primary RHMS phase	Who is being monitored?	Location of end‐user*	Health care focus	What is being monitored?	RHMS device components	Where does the data go?
Egede^104^	Quantitative	Deployment	Individual	Low‐income, rural population	Diabetes	Physiologic, blood glucose	Glucometer, wearable vital signs devices	Community hospital/Clinic
Eisenhauer^68^	Multi‐method	Deployment	Individual	Agriculture‐based, isolated rural county in the US Northern Plains state	Wellness/Prevention, obesity	Mobility/Activity, weight, nutrition	Watch wearable, wearable vital signs device	Self‐monitoring individual, research team
Hicken^107^	Quantitative	Deployment	Caregiver	Rural locations	Wellness/Prevention, caregiver support	Symptoms, quality of life	Computer or other device with internet, telephone if computer device not available	Health care provider, allied health professional
Vadheim^117^	Quantitative	Deployment	Individual	Towns in frontier counties of Southeastern Montana	Diabetes, obesity	Blood glucose, mobility/activity, nutrition	Technology with internet access; varied depending on local capabilities	Self‐monitoring individual, allied health professional, community hospital/clinic
Wakefield^88^	Quantitative	Deployment	Individual	Rural Midwestern Veterans Affairs Medical Centers	Diabetes, other chronic illnesses	Symptoms, behavior management	Wearable vital sign device, other RHMS for diabetes and heart failure (not specified)	Health care provider
Batsis^93^	Mixed methods	Design	Individual	Rural new Hampshire	Obesity	Physiologic, blood glucose, mobility/activity, symptoms, weight, nutrition, sleep	Watch wearable	Self‐monitoring individual, health care provider, research team
Bonsignore^63^	Mixed methods	Deployment	Individual and caregiver	Rural western North Carolina	Palliative care	Symptoms	Smartphone or tablet	Research team, palliative care clinic
Mallow^108^	Mixed methods	Design	Individual	Rural West Virginia	Non‐specified chronic illness	Physiologic, weight	Tablet, glucometer	Self‐monitoring individual, health care provider, research team
Bergloff^97^	Qualitative	Design	Individual	Rural clinics in Alaska, Idaho, Oregon, Montana, Washington, and Utah	Wellness/Prevention, diabetes, other chronic illness	Blood glucose	Insulin pump and continuous glucose monitoring	Self‐monitoring individual
Runkle^83^	Quantitative	Design	Individual	Rural western North Carolina	Pregnancy	Unclear	Smartphone, wearable mobile sensor	Unclear
Sharaievska^84^	Qualitative	Design	Other‐ parents and their children	Rural Appalachia	Wellness/Prevention	Mobility/Activity	Watch wearable	Self‐monitoring individual, research team
Stringer^86^	Mixed methods	Design	Individual	Rural areas of the United States Deep South	Mental health	Medication adherence	Electronic pill box	Self‐monitoring individual, health care provider, allied health professional, research team

* Per study author description.

**TABLE 5:** Summary of key study characteristics in remote health monitoring systems (RHMS) designed and/or deployed for rural participants, 2020–2022 (*n* = 28).

References	Primary study design*	Primary RHMS phase	Who is being monitored?	Location of end‐user*	Health care focus	What is being monitored?	RHMS device components	Where does the data go?
Beattie^100^	Quantitative	Design and deployment	Other‐ homes of older adults	Low‐income section 202 housing residents in Portland, African American and Latinx and Hispanic residents in Chicago and Miami, and veterans living in rural areas of Oregon and Washington	Wellness/Prevention	Physiologic, mobility/activity, weight, nutrition, sleep, social engagement, medication adherence	Watch wearable, sleep mat, driving sensors, smart device, digital scale, ambient/environmental sensors, electronic pill box	Research team
Davis^67^	Qualitative	Design	Individual	West Virginia	Non‐specified chronic illness	Physiologic, symptoms, weight	Remote monitoring devices (not specified)	Health care provider, allied health professional
Davis^15^	Quantitative	Deployment	Individual	Rural health clinic in north Mississippi	Diabetes	Blood glucose	Tablet, glucometer	Self‐monitoring individual, health care provider
Higa^72^	Multi‐method	Deployment	Individual	The island of Moloka‘i in the state of Hawaiʻi	Diabetes	Blood glucose	Smartphone, glucometer	Self‐monitoring individual and caregiver, health care provider, allied health professional
Ingram^73^	Multi‐method	Design	Individual and caregiver	Hospice patients admitted to the Mayo Clinic Health System Hospice in Eau Claire, Wisconsin	Hospice	Symptoms	Video monitoring platform	Self‐monitoring individual, caregiver, health care provider, allied health professional, research team
Polgreen^110^	Mixed methods	Design	Individual	Rural areas	Hypertension	Physiologic	Smartphone	Self‐monitoring individual, health care provider, research team
Yin^89^	Qualitative	Design	Individual	Rural South Texas	Obesity	Mobility/Activity, weight, nutrition, sleep	Watch wearable, smart device, digital scale	Self‐monitoring individual, health care provider, research team
Alexander^91^	Qualitative	Design	Individual	Rural Western North Carolina	Chronic obstructive pulmonary disease	Unclear	Remote patient monitoring devices (not specified)	Health care provider, unclear
Batsis^60^	Mixed methods	Deployment	Individual	Rural New Hampshire and Vermont	Wellness/Prevention, obesity	Mobility/Activity, nutrition	Watch wearable	Self‐monitoring individual, research team
Bechtel^18^	Mixed methods	Deployment	Individual	Rural patients from Arkansas, Washington, or Michigan	Mental health	Symptoms, cognition/mental status	Smartphone, unclear, application for any smart device	Self‐monitoring individual, health care provider, allied health professional
Clark^101^	Quantitative	Deployment	Individual	Rural and low‐income population	Hypertension	Physiologic	Tablet, wearable vital signs device	Allied health provider
Eisenhauer^69^	Multi‐method	Deployment	Individual	Northeast Nebraska	Wellness/Prevention, obesity	Mobility/Activity, weight, nutrition	Smartphone, digital scale	Self‐monitoring individual, research team
Nicosia^80^	Qualitative	Deployment	Individual	Rural Veterans	Obstructive sleep apnea	Sleep	Smartphone or tablet, sleep apnea testing system	Health care provider
Runkle^113^	Quantitative	Deployment	Individual	Western North Carolina	Hypertension in pregnancy	Physiologic	Smartphone, wearable vital signs device	Self‐monitoring individual, research team
Silfee^85^	Qualitative	Design	Individual	Both urban and rural patients	Mental illness	Symptoms	Smartphone	Self‐monitoring individual, health care provider, allied health professional
Aikens^99^	Mixed methods	Deployment	Individual	Patients with depression throughout rural, suburban and urban Michigan	Depression	Symptoms, medication adherence	Telephone	Self‐monitoring individual, caregiver health care provider, research team
Aronoff‐Spencer^92^	Quantitative	Design and deployment	Individual	Rural Kentucky, Appalachian Kentucky	Cancer	Symptoms	Application for any smart device	Self‐monitoring individual and caregiver, health care provider
Countouris^66^	Quantitative	Deployment	Individual	Western and Central Pennsylvania	Hypertension in pregnancy	Physiologic	Computer device (i.e., smartphone, tablet, computer)	Academic hospital/Clinic
Gorczyca^70^	Multi‐method	Deployment	Individual	Rural Kansas counties	Diabetes	Weight, nutrition	Watch wearable	Self‐monitoring individual, research team
LeBaron^16^, ^19^	Multi‐method	Deployment	Individual and caregiver	Central Virginia, some participants described as “rural residing”	Cancer	Physiologic, mobility/activity, symptoms, ambient/environment	Watch wearable, ambient/environmental sensors	Research team
Magnani^77^	Mixed methods	Deployment	Individual	Rural Western Pennsylvania	Atrial fibrillation	Physiologic, medication adherence	Smartphone	Self‐monitoring individual
Morgan^79^	Multi‐method	Design	Individual	Northern New England	Prenatal care	Physiologic, weight, fetal kick count	At‐home self‐monitoring tools: wearable vital signs devices, scale, Doppler	Self‐monitoring individual
Kobe^95^	Mixed methods	Deployment	Individual	Veterans Health Administration sites serving rural populations	Diabetes	Blood glucose	Glucometer	Self‐monitoring individual, health care provider
Li^76^	Mixed Methods	Design	Individual	Rural south Texas	Diabetes	Blood glucose, mobility/activity, weight, nutrition	Watch wearable, smart device	Self‐monitoring individual, research team
Vernon^87^	Qualitative	Design	Individual	A 13 county public health district in east Georgia	Postpartum	Physiologic, symptoms, weight	Smartphone or tablet	Self‐monitoring individual
Waddell^118^	Multi‐method	Design and deployment	Individual	Counties in Pennsylvania, Delaware, and parts of Ohio, New York, New Jersey, and West Virginia	Neurologic disease	Mobility/Activity	Fitbit, computer device (i.e., smartphone, tablet, computer)	Research team
Rowland^81^	Quantitative	Design	Individual	Rural Hispanic/Latino adults	Cardiometabolic health	Mobility/activity, weight, nutrition	Smartphone, digital scale	Self‐monitoring individual, health care provider, research team
Rowland^82^	Mixed methods	Design	Individual	Rural Hispanic/Latino adults	Cardiovascular risk	Mobility/Activity, weight, nutrition	Smartphone, digital scale	Self‐monitoring individual, health care provider, research team

* Per study author description.

**TABLE 6:** Summary of key study characteristics in remote health monitoring systems (RHMS) designed and/or deployed for rural participants, 2023–2024 (*n* = 11).

References	Primary study design*	Primary RHMS phase	Who is being monitored?	Location of end‐user*	Health care focus	What is being monitored?	RHMS device components	Where does the data go?
De Anda‐Duran^59^	Qualitative	Design	Individual	Rural area of Bogalusa, Louisiana	Cognitive impairment	Physiologic, mobility/activity, sleep, cognition/mental status	Watch wearable, other wearable, smartphone, wearable vital signs device	Self‐monitoring individual, allied health professional, research team, rural hospital/clinic
Durr^103^	Quantitative	Deployment	Individual	Rural West Virginia	Hypertension	Physiologic	Wearable vital signs device	Self‐monitoring individual, health care provider, allied health professional, research team
Kim^74^	Quantitative	Design and deployment	Individual	Rural and agricultural counties in California	Diabetes, hypertension	Physiologic, blood glucose	Tablet, glucometer, wearable vital signs devices	Health care provider, allied health professional
Kirkland^75^	Multi‐method	Deployment	Individual	Three contiguous rural counties	Diabetes, hypertension	Physiologic, blood glucose	2‐in‐1 blood glucose and blood pressure monitoring device	Research team, rural hospital/clinic, academic hospital/clinic
Mallow^78^	Multi‐method	Deployment	Individual	Rural West Virginia	Disability	Physiologic, blood glucose, weight, falls	Glucometer, wearable vital signs devices	Academic hospital/clinic
Rai^111^	Quantitative	Design	Individual	Diabetes patients in rural areas	Diabetes	Medication adherence	Smart device	Self‐monitoring individual, caregiver
Boente^62^	Multi‐method	Deployment	Individual	Rural regions served by the Indiana University ILD (interstitial‐lung disease) clinic	Interstitial lung disease	Physiologic, symptoms, quality of life	Smartphone or tablet	Self‐monitoring individual, unclear
Eberly^94^	Quantitative	Deployment	Individual	Rural Navajo Nation	Heart failure	Physiologic, medication adherence	Telephone	Research team, rural hospital/clinic
Grant^71^	Quantitative	Deployment	Individual	Rural areas of South Carolina	Hypertension	Physiologic	Wearable vital sign devices	Self‐monitoring individual, health care provider, allied health professional
Sulieman^116^	Quantitative	Deployment	Individual	Rural West Tennessee	Acute illness recovery	Physiologic	Smartphone	Community hospital/Clinic
Zhang^14^	Quantitative	Deployment	Individual	Rural Mississippi	Hypertension in pregnancy	Physiologic, symptoms	Tablet, wearable vital signs device	Research team, academic hospital/clinic

* Per study author description.

Our results are organized and presented below related to the key themes identified across our sample of 61 articles: (1) stakeholder engagement in RHMS development; (2) broadband and connectivity considerations; (3) use of formal frameworks to inform the research process; (4) cost considerations; (5) defining “rural”; (6) tailoring RHMS to rural contexts; and (7) author‐reported barriers and facilitators to RHMS use in rural settings, and (8) author‐reported study limitations. Additional components of RHMS are discussed in the following section. Key results are summarized in Table 7.

**TABLE 7:** Studies on remote health monitoring systems (RHMS) for rural participants that incorporated stakeholder engagement, financial considerations, broadband access, human/user‐centered or community‐based participatory design approaches, or an operational definition of rural.

	Engaged with stakeholders	Discussed financial considerations of RHMS	Discussed broadband and addressed barriers	Used human‐centered/user‐centered design principles	Used CBPR principles	Provided operational definition of rural
Alexander^91^	*	*				*
Aronoff‐Spencer^92^	*	*	*	*	*	*
Batsis^60^	*					
Batsis^93^	*			*		
Beattie^100^		*	*			
Bechtel^18^	*	*				
Bergloff^97^		*				
Boente^62^	*					*
Bonsignore^63^	*					*
Chen^64^	*	*	*			*
Cherry^65^	*		*			*
Clark^101^		*				*
Countouris^66^	*	*				
Davis^67^	*	*	*			*
Davis^15^		*	*			
De Anda‐Duran^59^	*		*			*
Durr^103^			*			*
Eberly^94^	*	*	*			
Egede^104^		*	*			
Eisenhauer^68^	*	*			*	*
Eisenhauer^69^	*					*
Foley^105^		*	*			
Frisbee^106^		*	*			
Giger^98^		*			*	*
Gorczyca^70^	*					*
Grant^71^	*	*	*			
Hicken^107^			*			*
Higa^72^	*	*			*	*
Ingram^73^	*					
Kim^74^	*		*		*	*
Kirkland^75^	*	*				*
Kobe^95^	*		*			*
LeBaron^16^, ^19^	*		*	*		
Li^76^	*	*				*
Magnani^77^	*	*	*			*
Mallow^78^	*	*	*			
Mallow^108^		*	*			
Morgan^79^	*	*				*
Nicosia^80^	*					*
Peng^96^	*					
Petitte^109^			*			
Polgreen^110^		*	*			*
Rai^111^						
Riley^112^		*	*			
Rowland^81^	*					
Rowland^82^	*				*	*
Runkle^83^	*	*				*
Runkle^113^						*
Shane‐McWhorter^114^			*			
Sharaievska^84^	*					*
Silfee^85^	*	*				
Stringer^86^	*					
Vadheim^117^		*	*			*
Vernon^87^	*					*
Waddell^118^			*			*
Wakefield^88^	*	*				
Yin^89^	*		*		*	
Zhang^14^	*					
Total *n* (%) out of 61 studies (*n* = 61; 100%)	39 (63.9%)	30 (49.2%)	27 (44.3%)	3 (4.9%)	7 (11.5%)	31 (50.8%)

Abbreviation: CBPR, community‐based participatory research.

* The study included or demonstrated the specified feature.

### Theme 1: Stakeholder engagement in RHMS development

Of the total 61 studies, the majority (63.9%, *n* = 39) reported some degree of stakeholder engagement (i.e., input from invested parties, such as patients) (see Table 7), although the type of stakeholder, timing, and scope of involvement varied. Of these 39 studies, stakeholders were most commonly engaged during the deployment phases of study (84.6%, *n* = 33)^16^, ^61^, ^62^, ^63^, ^64^, ^65^, ^66^, ^67^, ^68^, ^69^, ^70^, ^71^, ^72^, ^73^, ^74^, ^75^, ^76^, ^77^, ^78^, ^79^, ^80^, ^81^, ^82^, ^83^, ^84^, ^85^, ^86^, ^87^, ^88^, ^89^, ^90^, ^91^, ^92^; fewer studies incorporated stakeholders during the design phase, prior to deploying the RHMS to end‐users (59%, *n* = 23).^16^, ^61^, ^62^, ^66^, ^69^, ^74^, ^75^, ^77^, ^80^, ^83^, ^84^, ^85^, ^86^, ^87^, ^88^, ^89^, ^91^, ^93^, ^94^, ^95^, ^96^, ^97^, ^98^

Of the 39 studies that engaged stakeholders, patients were the most commonly engaged, with 82.1% (*n* = 32) studies reporting patient participation.^16^, ^61^, ^62^, ^63^, ^64^, ^65^, ^66^, ^67^, ^68^, ^69^, ^70^, ^71^, ^72^, ^74^, ^77^, ^78^, ^79^, ^80^, ^81^, ^82^, ^83^, ^84^, ^85^, ^86^, ^88^, ^89^, ^91^, ^92^, ^94^, ^95^, ^96^, ^98^ Other stakeholder groups included health care providers (43.6%, *n* = 17),^16^, ^63^, ^65^, ^69^, ^71^, ^74^, ^75^, ^76^, ^80^, ^84^, ^85^, ^87^, ^89^, ^94^, ^95^, ^96^, ^97^ and community members (30.8%, *n* = 12).^16^, ^71^, ^73^, ^75^, ^78^, ^83^, ^84^, ^91^, ^93^, ^94^, ^95^, ^96^ Of the 39 studies that engaged stakeholders,, six (15.4%, *n* = 6) studies included family/informal caregivers in their stakeholder groups.^65^, ^67^, ^74^, ^77^, ^90^, ^94^ Some studies engaged other relevant stakeholders, such as health care administrators (7.7%, *n* = 3),^69^, ^75^, ^80^ content experts (7.7%, *n* = 3),^69^, ^75^, ^80^ and RHMS vendors (5.1%, *n* = 2).^69^, ^75^

### Theme 2: Broadband and connectivity considerations

To fully capture how studies addressed broadband access, we considered the stage of development: studies early in the design phase (e.g., stakeholder interviews, needs assessments) were examined for whether broadband challenges were acknowledged, whereas studies further along in the design or deployment phase were evaluated for whether and how the RHMS was adapted to account for limited broadband.

Out of the total 61 studies, 13.1% (*n* = 8) were in earlier stages of RHMS design, and they did not report intention or plans to adapt the RHMS for limited broadband access.^63^, ^82^, ^87^, ^89^, ^93^, ^98^, ^99^, ^100^ Of these eight studies, 50% (*n* = 4) acknowledged potential concerns about broadband or internet access,^63^, ^89^, ^93^, ^99^ and 50% (*n* = 4) did not discuss broadband access.

Out of the total 61 studies, 86.9% (*n* = 53)^16^, ^61^, ^62^, ^64^, ^65^, ^66^, ^67^, ^68^, ^69^, ^70^, ^71^, ^72^, ^73^, ^74^, ^75^, ^76^, ^77^, ^78^, ^79^, ^80^, ^81^, ^83^, ^84^, ^85^, ^86^, ^88^, ^90^, ^91^, ^92^, ^94^, ^95^, ^96^, ^97^, ^101^, ^102^, ^103^, ^104^, ^105^, ^106^, ^107^, ^108^, ^109^, ^110^, ^111^, ^112^, ^113^, ^114^, ^115^, ^116^, ^117^, ^118^, ^119^, ^120^ were further along in design and/or deployment. Ofthese 53 studies, the authors either (1) identified broadband or internet‐related concerns and adapted their RHMS accordingly (see Table 7); (2) identified broadband concerns but excluded participants from the study who did not have reliable internet access; (3) identified these broadband concerns but did not state whether adaptations were made (20.8%; *n* = 11)^62^, ^64^, ^65^, ^70^, ^72^, ^74^, ^81^, ^84^, ^92^, ^95^, ^118^; or (4) did not discuss broadband concerns at all (18.9%; *n* = 10).^68^, ^75^, ^78^, ^83^, ^85^, ^86^, ^90^, ^103^, ^113^, ^117^ Of the total studies, 3.8% (*n* = 2)^71^, ^115^ studies stated that all participants had broadband access, and 5.7% (*n* = 3)^76^, ^88^, ^101^ of studies stated that broadband was not required for system functionality.

Adaptations to broadband challenges varied. Some studies provided data plans or cellular‐enabled devices.^16^, ^66^, ^67^, ^77^, ^79^, ^102^, ^104^, ^108^, ^110^ Others designed applications to work in low‐connectivity environments.^91^, ^94^ Specific strategies to reduce high‐speed, high‐capacity internet requirements included pre‐downloaded content^91^; use of telephone‐based check‐ins instead of video conferencing^70^, ^71^, ^93^, ^94^, ^103^, ^104^, ^106^, ^109^, ^113^; systems compatible with landlines^66^, ^67^, ^80^, ^88^, ^97^, ^106^, ^111^; or use of Short Message Service (text messaging).^107^, ^112^ Across all studies, 16.4% (*n* = 10) excluded participants without reliable internet access.^62^, ^64^, ^65^, ^72^, ^78^, ^84^, ^92^, ^102^, ^113^, ^118^

### Theme 3: Use of formal frameworks to inform the research process

Formal frameworks were rarely cited. Across all studies, 4.9% (*n* = 3) studies referenced human‐centered design/user‐centered design (see Table 7). A greater number of studies (11.5%, *n* = 7) applied community‐based participatory research (CBPR) principles (see Table 7). However, the majority of studies (83.6%, *n* = 51)^61^, ^62^, ^64^, ^65^, ^66^, ^67^, ^68^, ^69^, ^72^, ^73^, ^75^, ^76^, ^78^, ^79^, ^80^, ^81^, ^82^, ^83^, ^85^, ^86^, ^87^, ^88^, ^89^, ^90^, ^92^, ^93^, ^96^, ^97^, ^98^, ^99^, ^101^, ^102^, ^103^, ^104^, ^105^, ^106^, ^107^, ^108^, ^109^, ^110^, ^111^, ^112^, ^113^, ^114^, ^115^, ^116^, ^117^, ^118^, ^119^, ^120^ did not clearly indicate the use of any of these guiding frameworks.

### Theme 4: Cost considerations

Financial considerations related to RHMS were variably addressed. Although 49.2% (*n* = 30) studies discussed costs associated with RHMS (see Table 7), 45.9% (*n* = 28) did not,^16^, ^61^, ^62^, ^64^, ^65^, ^67^, ^71^, ^72^, ^75^, ^77^, ^82^, ^83^, ^84^, ^86^, ^88^, ^89^, ^91^, ^95^, ^98^, ^101^, ^109^, ^111^, ^113^, ^115^, ^116^, ^117^, ^118^, ^120^ and 1.6% (*n* = 1)^97^ study explicitly stated that cost was irrelevant to system function. Among those that did address financial considerations, discussions ranged from identifying cost as a barrier to RHMS—due to limited insurance coverage or low‐income in rural populations and high device or data costs^63^, ^70^, ^76^, ^78^, ^81^, ^87^, ^93^, ^94^, ^99^, ^100^, ^102^, ^103^, ^107^, ^110^—to emphasizing the potential for RHMS to be cost‐effective, lower travel expenses, and ultimately reduce health care expenditures if integrated with reimbursement policies.^66^, ^73^, ^76^, ^85^, ^87^, ^100^, ^106^, ^110^, ^119^ Of all studies, 19.7% (*n* = 12) studies highlighted strategies to reduce costs through community partnerships,^74^, ^94^ use of low‐cost or scalable technologies (e.g., text messaging, telephone),^70^, ^94^, ^96^, ^102^, ^107^, ^112^ and grant or insurance reimbursement support.^73^, ^96^, ^100^, ^108^

### Theme 5: Defining “rural”

Approximately half (50.8%, *n* = 31) of studies provided an operational definition of “rural,” but the definition used was highly variable (see Table 7). Of the 30 studies that provided an operational definition of rural, 35% (*n* = 7) used Rural‐Urban Commuting Areas^70^, ^71^, ^82^, ^84^, ^97^, ^112^, ^120^; 5% (*n* = 1) used the Federal Office of Rural Health Policy^79^; 5% (*n* = 1) used Rural Continuum Codes^81^; 5% (*n* = 1) used Urban Areas and Urban Clusters^67^; 5% (*n* = 1) used Frontier and Remote Access Codes^119^; 5% (*n* = 1) used the Patient Protection and Affordable Care Act definition^100^; 5% (*n* = 1) used the National Center for Health Statistics Urban–Rural Scheme^64^; and 5% (*n* = 1) used Veterans Affairs rurality algorithm^109^. Of the 29 studies that defined rurality at the geographic level, 17.2% (*n* = 5) were defined at the county level^61^, ^70^, ^78^, ^84^, ^100^; 13.8% (*n* = 4) used the US Census Tract^67^, ^79^, ^100^, ^120^; 17.2% (*n* = 5) provided rural zip codes^64^, ^97^, ^105^, ^109^, ^112^; and 10.3% (*n* = 3) specified a rural region (e.g., Appalachia).^86^, ^115^, ^119^ In contrast, 49.2% (*n* = 30) of all 61 studies mentioned rurality without a clear formal definition.^62^, ^63^, ^68^, ^73^, ^75^, ^77^, ^80^, ^83^, ^87^, ^88^, ^90^, ^91^, ^92^, ^93^, ^95^, ^96^, ^98^, ^99^, ^101^, ^102^, ^104^, ^106^, ^107^, ^108^, ^110^ ^,^ ^111^, ^113^, ^114^, ^116^, ^117^, ^118^

### Theme 6: Tailoring RHMS to rural contexts

Out of all 61 studies, 29.5% (*n* = 18) studies considered distance to care when designing the RHMS.^64^, ^67^, ^68^, ^74^, ^75^, ^78^, ^82^, ^87^, ^94^, ^100^, ^105^, ^109^, ^110^, ^112^, ^117^, ^119^, ^120^ Some studies addressed additional contextual factors, including digital literacy‐ the skills to use digital technologies effectively (27.9%, *n* = 17),^16^, ^61^, ^62^, ^67^, ^71^, ^78^, ^79^, ^83^, ^84^, ^87^, ^91^, ^98^, ^101^, ^105^, ^107^, ^108^, ^110^ privacy and security concerns (13.1%, *n* = 8),^61^, ^77^, ^81^, ^82^, ^91^, ^93^, ^98^, ^102^ and community distrust in health care systems (11.5%, *n* = 7).^72^, ^74^, ^78^, ^82^, ^89^, ^105^, ^110^ Other studies incorporated additional rural‐relevant adaptations, such as accounting for low health literacy—the ability to understand and use health information^69^, ^74^, ^78^, ^79^; designing smaller or less obtrusive wearables to match the preferences of their physically active, rural male population^70^; developing RHMS that worked effectively on limited‐capability smartphones^63^, ^76^; and simplifying systems to reduce digital burden, particularly for those with low digital literacy.^61^, ^101^ Although RHMS reduced transportation demands, some in‐person visits remained necessary for health or study purposes. Certain studies mitigated this burden by limiting required visits or localizing interventions, an approach particularly helpful for rural participants.^67^, ^95^, ^105^ Recognizing that rural populations in the US have higher rates of disability, which can create sensory or medical barriers to technology use, one study incorporated feedback from rural end‐users to design an RHMS that was accessible and usable despite these challenges.^95^

In addition, we extracted data pertaining to barriers and facilitators influencing RHMS design and/or deployment, as well as study limitations, identified or discussed by study authors or participants. These barriers, facilitators, and limitations are summarized below.

### Theme 7: Author‐reported barriers and facilitators to RHMS use in rural settings

#### Author‐reported barriers to RHMS use in rural settings

Of all studies, 68.9% (*n* = 42) studies identified a broad range of barriers to RHMS use for rural residents.^16^, ^61^, ^62^, ^63^, ^64^, ^66^, ^67^, ^68^, ^69^, ^70^, ^72^, ^73^, ^74^, ^75^, ^76^, ^77^, ^78^, ^79^, ^80^, ^81^, ^82^, ^83^, ^85^, ^86^, ^87^, ^88^, ^89^, ^91^, ^93^, ^96^, ^99^, ^102^, ^104^, ^105^, ^108^, ^110^, ^112^, ^113^, ^115^, ^116^, ^118^, ^120^ Limited broadband access^63^, ^64^, ^70^, ^73^, ^74^, ^81^, ^89^, ^93^, ^102^, ^105^, ^118^ and cost emerged as recurring barriers, with many participants unable to afford the necessary devices or data plans.^61^, ^70^, ^74^, ^87^, ^118^, ^120^ Challenges with technology performance and ease of use were common and included application (“app”) malfunctions or limitations and confusing interfaces.^57^, ^61^, ^68^, ^72^, ^83^, ^102^ Active monitoring (e.g., participants needing to actively engage with a system to enter data) created participant burden, which was identified as a barrier,^67^, ^70^, ^74^, ^80^, ^108^, ^116^ whereas passive monitoring raised privacy and confidentiality concerns, especially when environmental sensors or GPS data were involved.^85^, ^115^, ^118^ Many study participants reported limited digital literacy or discomfort with unfamiliar devices,^61^, ^73^, ^78^, ^79^, ^83^, ^118^ and some participants expressed skepticism about the benefits or necessity of RHMS.^63^, ^69^, ^85^ Other issues included limited clinician time^63^, ^85^, ^96^ and insufficient training on RHMS devices for patients and/or providers.^74^, ^83^, ^99^ Cultural factors, including preferences for in‐person care^68^, ^87^, ^93^ and language barriers,^68^, ^73^, ^116^ also limited engagement. Retention challenges and high dropout rates limited scalability.^66^, ^73^, ^76^, ^116^

#### Author‐reported facilitators of RHMS use in rural settings

Of all studies, 82% (*n* = 50) studies identified key facilitators to successful RHMS use for rural residents.^16^, ^61^, ^62^, ^64^, ^65^, ^66^, ^67^, ^68^, ^69^, ^70^, ^71^, ^72^, ^73^, ^74^, ^75^, ^76^, ^77^, ^78^, ^79^, ^81^, ^82^, ^83^, ^84^, ^85^, ^86^, ^87^, ^88^, ^89^, ^93^, ^94^, ^96^, ^97^, ^99^, ^100^, ^101^, ^102^, ^103^, ^104^, ^105^, ^107^, ^108^, ^109^, ^110^, ^111^, ^113^, ^115^, ^116^, ^117^, ^119^, ^120^ Providing devices, such as smartphones or tablets, or providing data plans at no cost emerged as a powerful facilitator,^16^, ^66^, ^67^, ^69^, ^77^, ^79^, ^91^, ^102^, ^104^, ^107^, ^108^, ^110^, ^111^ enabling participation among individuals who otherwise lacked access. Designing RHMS that did not require consistent broadband connectivity,^91^, ^94^, ^102^, ^105^, ^112^, ^120^ or offering alternative modalities like telephone‐based care,^61^, ^66^, ^73^, ^80^, ^88^, ^96^, ^107^, ^109^, ^116^, ^119^ helped accommodate infrastructural constraints. Some studies offered technical support or pre‐installed equipment to reduce mental burden.^91^, ^102^, ^104^ Language‐congruent platforms and bilingual staff helped address communication barriers and increase trust.^16^, ^73^, ^83^, ^84^, ^96^, ^116^ There were 13.1% (*n* = 8) studies that emphasized the value of training—particularly in‐person training prior to deployment—which improved participant confidence and clinician buy‐in.^73^, ^75^, ^76^, ^83^, ^87^, ^97^, ^104^, ^110^ There were 16.4% (*n* = 10) studies that reported that RHMS participation that enhanced patient awareness, confidence, and adherence led to greater retention.^16^, ^62^, ^70^, ^86^, ^87^, ^88^, ^93^, ^103^, ^107^, ^117^ A sense of connection—whether with a care team, peers, or family—was often cited as a positive outcome that motivated participants to continue to use the RHMS.^61^, ^70^, ^73^, ^74^, ^76^, ^86^, ^105^, ^119^

Some studies highlighted community engagement as a critical factor (13.1%, *n* = 8).^16^, ^68^, ^70^, ^71^, ^74^, ^91^, ^93^, ^94^ RHMS codesigned with the rural community or adapted on the basis of local input were well‐aligned with user preferences and highly acceptable to rural populations.^16^, ^68^, ^70^, ^71^, ^74^, ^81^, ^91^, ^93^, ^94^ Community health workers and peer support mechanisms helped bridge gaps in literacy, trust, and cultural relevance.^73^, ^78^ Programs that incorporated family members or social supports,^74^, ^78^ or leaned into existing community strengths,^74^, ^94^, ^96^ were particularly well‐received. When interventions were culturally and linguistically tailored, recruitment and engagement were high.^16^, ^69^, ^71^, ^73^, ^74^, ^78^, ^84^, ^96^, ^116^

Ease of use was another consistent facilitator. RHMS devices with intuitive interfaces,^64^, ^65^, ^67^, ^77^, ^82^, ^111^ minimal user burden,^65^, ^69^, ^73^, ^77^, ^82^, ^87^, ^101^, ^105^, ^110^, ^120^ or multiple feature options^73^, ^102^—like the ability to use a smartphone, computer, or the telephone—were well‐accepted by rural participants with varying degrees of digital literacy. Participants in remote areas highly valued RHMS that eased transportation challenges and improved access to continuous care.^16^, ^61^, ^65^, ^66^, ^67^, ^68^, ^69^, ^73^, ^81^, ^82^, ^87^, ^97^, ^101^, ^103^, ^104^, ^105^, ^110^, ^111^, ^115^, ^117^, ^120^ When structured around evidence‐based protocols for clinical care of their respective patient populations,^69^, ^104^, ^110^ reimbursement mechanisms (i.e., private or government‐funded insurance coverage),^68^, ^74^, ^76^, ^78^, ^96^, ^104^, ^108^ and adapted to clinician schedules and preferences,^63^, ^76^, ^87^, ^99^, ^105^ RHMS interventions demonstrated scalability and potential for integration into standard workflows.

Studies that used ongoing monitoring and adaptation strategies, such as mentorship, real‐time feedback, or local adjustments, successfully managed emerging challenges and sustained engagement.^61^, ^63^, ^69^, ^71^, ^73^, ^75^, ^91^, ^97^ These customizations involved tailoring the RHMS to fit the unique needs, workflows, and constraints of specific clinical settings and rural populations.^61^, ^63^, ^69^, ^71^, ^73^, ^75^, ^91^, ^97^ Leadership training and consistent communication between staff and participants further strengthened the use of RHMS.^61^, ^75^, ^76^

### Theme 8: Author‐reported study limitations

Out of all 61 studies, 85.3% (*n* = 52) self‐acknowledged (e.g., identified by authors of the reviewed study, not the authors of this paper) important limitations.^16^, ^61^, ^62^, ^63^, ^64^, ^65^, ^66^, ^67^, ^68^, ^70^, ^71^, ^72^, ^74^, ^75^, ^76^, ^77^, ^78^, ^79^, ^80^, ^81^, ^82^, ^83^, ^84^, ^85^, ^86^, ^87^, ^88^, ^91^, ^93^, ^94^, ^95^, ^96^, ^97^, ^99^, ^100^, ^101^, ^102^, ^103^, ^104^, ^105^, ^106^, ^107^, ^108^, ^109^, ^110^, ^111^, ^113^, ^115^, ^116^, ^118^, ^119^, ^120^ The most frequent limitation 27.9% (*n* = 17) was small sample size, which restricted the statistical power and generalizability of findings.^16^, ^63^, ^65^, ^66^, ^67^, ^77^, ^78^, ^79^, ^81^, ^84^, ^96^, ^97^, ^100^, ^110^, ^113^, ^115^, ^120^

Out of all 61 studies, there were numerous studies (39.3%, *n* = 24) that identified their concentration on a specific rural patient population as a limitation to generalizability.^16^, ^41^, ^60^, ^62^, ^65^, ^67^, ^68^, ^69^, ^76^, ^78^, ^82^, ^88^, ^90^, ^92^, ^94^, ^97^, ^98^, ^99^, ^102^, ^105^, ^107^, ^110^, ^112^, ^117^ Despite intentions to achieve diverse representation, a subset of studies (19.7%, *n* = 12) ultimately included samples that were largely white, insured, and highly educated.^62^, ^63^, ^71^, ^72^, ^79^, ^81^, ^93^, ^95^, ^100^, ^102^, ^105^, ^120^ Additionally, 13.1% (*n* = 8) study authors reported that their interventions were limited to a single community or patients of a single health care site or system.^61^, ^65^, ^68^, ^74^, ^96^, ^106^, ^113^, ^118^ Several studies (11.5%, *n* = 7) depended on resources not broadly accessible, including access to clinicians with specialized expertise (e.g., maternal–fetal medicine, cardiology, or bilingual capability), as well as grant‐funded technology.^67^, ^68^, ^75^, ^77^, ^82^, ^96^, ^97^

Convenience sampling and recruitment biases, such as enrolling only participants who had already engaged with RHMS (either in a clinical setting or prior research study), also introduced potential selection bias.^63^, ^75^, ^81^, ^82^, ^85^, ^86^, ^87^, ^93^, ^110^, ^116^ Out of the entire sample (*n* = 61), some studies (19.7%, *n* = 12) discussed how they lacked control groups, randomization, or blinding, further reducing the strength of causal inference.^62^, ^72^, ^76^, ^100^, ^101^, ^103^, ^104^, ^109^, ^110^, ^115^, ^116^, ^119^ Inadequate follow‐up time,^68^, ^72^, ^86^, ^96^, ^106^, ^116^ high dropout rates,^16^, ^66^, ^86^, ^111^, ^113^, ^116^, ^118^ or premature trial termination due to funding constraints^106^ also limited many findings. A few studies (4.9% *n* = 3) noted that the timing of COVID‐19 may have affected results, either by inflating engagement with RHMS during lockdowns or complicating recruitment and data collection.^16^, ^87^, ^118^

## DISCUSSION

The findings reveal substantial variability in how studies defined rurality, engaged stakeholders, and incorporated considerations of digital infrastructure and formal design frameworks. Although RHMS were widely recognized as promising tools to mitigate rural health disparities, many RHMS designs and/or deployments did not fully account for the unique cultural and structural complexities of rural communities. The absence of a standardized definition of “rural” reflects broader challenges in rural health research, as it complicates comparisons across studies and influences which populations and contexts are represented^121^—and, by extension, may have affected which studies were identified and included in this review. Our results align with prior studies showing that RHMS can enhance access and continuity of care for rural residents—but only when infrastructural and contextual barriers are directly addressed.^5^, ^22^, ^30^, ^122^ Additionally, the limited and inconsistent application of structured design frameworks contrasts with the growing body of literature advocating for user‐centered and participatory approaches in digital health.^123^, ^124^, ^125^, ^126^ Although some studies applied principles of CBPR (see Table 7), few systematically used formal design methods, underscoring a gap between recommended best practices in the literature and real‐world application. Three key themes emerged from our analysis: (1) limited stakeholder engagement, (2) ongoing infrastructure barriers, and (3) significant variability in how the cultural and contextual considerations of rural patients are incorporated into RHMS design and deployment.

Out of all 61 studies, stakeholder engagement was reported in 63.9% (*n* = 39 studies; see Table 7); however, the type of stakeholder, timing, and scope of involvement varied. Patients were the most frequently involved group, but informal caregivers were included in only six studies.^65^, ^67^, ^74^, ^77^, ^90^, ^94^. This limited inclusion of caregivers may reflect several documented recruitment challenges, including caregivers’ time constraints and the tendency for RHMS to be designed primarily around patient needs rather than caregiver involvement.^127^, ^128^, ^129^, ^130^, ^131^, ^132^, ^133^, ^134^, ^135^, ^136^ As a result, caregivers may perceive limited relevance or benefit to the use of RHMS or participation in studies related to RHMS, contributing to lower interest or engagement.^136^

Informal caregivers often perform a central role in patient care,^132^ including patient medication adherence,^130^, ^137^ tracking and managing patient symptoms,^129^, ^131^ coordinating health care appointments,^136^, ^138^ participating in treatment decision‐making,^131^, ^133^, ^134^ and providing emotional support and advocacy.^131^, ^132^, ^138^ Given their large role in supporting patients, when caregivers are underrepresented in the design and deployment of RHMS, these technologies may have limited utility and relevance in real‐world settings. Therefore, despite challenges in recruitment,^127^, ^128^, ^135^ including caregiver perspectives is critical to ensuring RHMS meet the needs of rural residents.^132^

Broadband access is foundational for RHMS functionality,^30^, ^139^, ^140^ yet only half of all studies explicitly addressed this critical topic (see Table 7). Among the studies that acknowledged connectivity challenges, strategies to overcome these challenges varied widely. For studies where broadband access was not explicitly addressed, it was sometimes unclear if broadband capability was assessed prior to designing or deploying the RHMS or if broadband access simply was not a problem and therefore not mentioned in the reporting. These findings are consistent with existing literature that considerations for inadequate broadband are limited, highlighting persistent digital health inequities and unmet infrastructure needs in rural settings.^141^, ^142^, ^143^ Adapting to limited broadband access may require significant changes to an RHMS, which may not always be technically feasible.^142^, ^144^ Such challenges may lead to RHMS being designed primarily for patients with adequate internet access, thereby excluding those in more rural or remote areas who already face significant barriers to health care access.^30^, ^139^, ^140^, ^141^, ^142^, ^143^, ^144^

Contextual considerations for the design and deployment of RHMS for rural residents varied. Although some interventions integrated bilingual interfaces to accommodate the growing number of non‐English speaking rural residents,^16^, ^73^, ^83^, ^84^, ^96^, ^116^, few addressed broader cultural considerations, such as privacy concerns, distrust of health care systems, or distance to care that have been documented as common concerns in rural areas.^37^, ^38^, ^39^, ^40^ Some studies considered health literacy^69^, ^74^, ^78^, ^79^ and/or digital literacy^16^, ^61^, ^62^, ^67^, ^71^, ^78^, ^79^, ^83^, ^84^, ^87^, ^91^, ^98^, ^101^, ^105^, ^107^, ^108^, ^110^; however, 68.9% (*n* = 42) studies did not reference or consider either form of literacy.^63^, ^64^, ^65^, ^66^, ^68^, ^71^, ^72^, ^73^, ^75^, ^76^, ^77^, ^80^, ^81^, ^82^, ^85^, ^86^, ^88^, ^89^, ^90^, ^92^, ^93^, ^94^, ^95^, ^96^, ^97^, ^99^, ^100^, ^102^, ^103^, ^104^, ^106^, ^109^, ^111^, ^112^, ^113^, ^114^, ^115^, ^116^, ^117^, ^118^, ^119^, ^120^ The use of formal frameworks was rare, with only three studies citing human‐ or user‐centered design and seven referencing using CBPR principles (see Table 7). The studies that did not use these frameworks may have overlooked key user needs, potentially limiting the accessibility and effectiveness of their RHMS.^124^, ^125^, ^126^ Although many authors viewed the narrow focus of their interventions—such as targeting a specific rural community—as a limitation,^16^, ^41^, ^60^, ^62^, ^65^, ^67^, ^68^, ^69^, ^76^, ^78^, ^82^, ^88^, ^90^, ^92^, ^94^, ^97^, ^98^, ^99^, ^102^, ^105^, ^107^, ^110^, ^112^, ^117^ one could argue that this approach is a strength, allowing for more tailored, context‐specific solutions rather than relying on a one‐size‐fits‐all model.^8^, ^14^, ^29^, ^30^, ^31^, ^37^, ^40^, ^43^, ^145^

These findings highlight several policy priorities for improving RHMS design and deployment in rural communities. Reliable broadband should be recognized as essential health care infrastructure, with expanded investment through federal and state initiatives, such as the Federal Communications Commission's Rural Healthcare Program^146^ and the Broadband Equity, Access, and Deployment Program to close persistent connectivity gaps and ensure equitable participation in RHMS.^147^ Future policy efforts should strengthen Centers for Medicare & Medicaid Services reimbursement pathways for RHMS services, including coverage for technology and connectivity.^148^, ^149^ Expanding reimbursement models to recognize RHMS as a reimbursable service could incentivize adoption by rural health care providers and sustain long‐term use.^36^ Federal and state funding agencies—such as the Health Resources and Services Administration—should prioritize RHMS research that evaluates cost‐effectiveness and long‐term integration into rural clinical systems.^150^

A key priority for future research in RHMS design is the proactive assessment of broadband access prior to deployment, enabling appropriate technological adaptations to ensure maximum accessibility across rural contexts.^30^, ^139^, ^140^ Engaging caregivers as stakeholders in RHMS design and deployment represents a critical opportunity to leverage their unique insights and improve the effectiveness of these interventions for real‐world caregiving needs.^129^, ^131^, ^136^ Future studies should aim to include more diverse rural populations and address intersectional disparities related to race, socioeconomic status, and geography.^151^ Examining privacy, data security, and trust dynamics remains essential, particularly in rural communities with historical health care mistrust.^27^, ^33^, ^34^ Finally, future research should utilize standardized frameworks for RHMS design and deployment, rooted in user‐centered and participatory principles and responsive to contextual needs and preferences.^123^, ^124^, ^125^, ^126^

### Limitations

We restricted our review to studies published in English and conducted in the US, which may limit generalizability to other global rural contexts. Additionally, the protocol for this scoping review was not prospectively registered in a public database, which may limit transparency and reproducibility. We did not appraise the quality of included studies; this is an opportunity for future research as the nature and purpose of a scoping review is to provide a broad overview of diverse evidence.^152^, ^153^ Although this review was guided by Arksey and O'Malley's original framework, we did not formally consult the PRISMA‐ScR checklist^154^ or the JBI scoping review guidelines,^155^ which provide updated extensions of this approach.^156^ As a result, some reporting elements emphasized in the PRISMA‐ScR or JPI frameworks may not have been fully addressed. Additionally, although consultation with stakeholders is identified as an optional component of the Arksey and O'Malley framework, this step was not included in the current review because of time and resource constraints and limited feasibility of capturing such a diverse range of stakeholders through the US; this is an opportunity for future research.^157^ It is possible that some included studies considered rural context in their design or deployment but did not explicitly report these details in their publications and thus are not included in our review. Inconsistent terminology for RHMS and rurality may have resulted in omission of relevant literature; we attempted to mitigate this by developing a comprehensive search strategy with the assistance of a health sciences librarian. Lastly, although every effort was made to ensure a comprehensive and objective review, we may have inadvertently missed or misinterpreted some study details.

## CONCLUSION

This scoping review provides a robust synthesis of recent evidence on RHMS design and deployment in rural US settings. RHMS have significant potential to reduce rural health disparities, but current research highlights gaps in their design and deployment for rural patients. Challenges include inconsistent definitions of rurality, limited stakeholder engagement, particularly of informal caregivers, variable attention to digital infrastructure, such as broadband access, and infrequent use of formal or participatory design frameworks. Future RHMS interventions for rural populations should proactively assess broadband access, meaningfully include caregivers and diverse rural populations, and adopt standardized, evidence‐based frameworks to guide RHMS design and deployment.

## CONFLICT OF INTEREST STATEMENT

The authors report no conflicts of interest.

## FUNDING INFORMATION

The authors declare that they have no funding disclose.

## Supporting information



Supporting Information

Supporting Information

## Data Availability

The data that support the findings of this study are available from the corresponding author upon reasonable request.
