# Supplementary material for: Rural community engagement and cultural considerations in the design and deployment of remote health monitoring systems: A scoping review
Source: J Rural Health. 2026 Apr 7;42(2):e70141. doi: 10.1111/jrh.70141 (PMC13058512; doi:10.1111/jrh.70141)
Supplement: Supplementary file 1 — Supporting Information [file JRH-42-0-s001.docx]

**Supplementary Data File 1.** PubMed, CINAHL, and Web of Science search strategies used in scoping review to explore RHMS designed and deployed for rural end-users.

For PubMed, the search strategy used was:

(("telemonitor*"[Title/Abstract] OR “telehome*”[Title/Abstract] OR ("monitor*"[Title/Abstract] AND ("remote"[Title/Abstract] OR “digital*”[Title/Abstract] OR “technolog*”[Title/Abstract] OR "telemedicine"[Title/Abstract] OR "telehealth"[Title/Abstract] OR "physiolog*"[Title/Abstract]))) AND ("rural"[Title/Abstract] OR "rurality"[Title/Abstract] OR "rural population"[MeSH Terms] OR "rural health"[MeSH Terms])) AND (english[Filter]). For CINAHL and Web of Science, the search terms used were: ((telemonitor* OR telehome*) OR (monitor AND (remote OR digital* OR technolog* OR telemedicine OR telehealth OR physiolog*))) AND (rural OR rurality OR “rural population” OR “rural health”).

For Web of Science (Core Collection), the search strategy used was:

((telemonitor* OR telehome*) OR (monitor AND (remote OR digital* OR technolog* OR telemedicine OR telehealth OR physiolog*))) AND (rural OR rurality OR “rural population” OR “rural health”)

For CINAHL Complete (accessed through EBSCO), the search strategy used was:

((telemonitor* OR telehome*) OR (monitor AND (remote OR digital* OR technolog* OR telemedicine OR telehealth OR physiolog*))) AND (rural OR rurality OR “rural population” OR “rural health”)

Searches in CINAHL Complete and Web of Science were conducted using keywords in titles and abstracts and, where applicable, database-specific subject headings.
