# Supplementary material for: Rural community engagement and cultural considerations in the design and deployment of remote health monitoring systems: A scoping review
Source: J Rural Health. 2026 Apr 7;42(2):e70141. doi: 10.1111/jrh.70141 (PMC13058512; doi:10.1111/jrh.70141)
Supplement: Supplementary file 2 — Supporting Information [file JRH-42-0-s002.docx]

**Supplementary Data File 2.** Extracted study characteristics and RHMS features with complete list of potential responses

| **Study Design*†** | **Primary RHMS Phase*** | **Healthcare focus*** | **What is, or is intended to be, measured/ monitored?*** | **Who or what is/is intended to be measured/monitored?*** | **RHMS device components*** | **Where does/will the data go?*** |
| --- | --- | --- | --- | --- | --- | --- |
| - Quantitative - Qualitative - Mixed methods - Multiple methods | - Planning/design - Deployment/ testing | - Wellness/ prevention - Diabetes - Hypertension - Cancer - Other chronic illness - Non-specified chronic illness - Acute illness/ recovery - Obesity - Mental health - Other (describe) | - Blood pressure - Heart rate - Respiratory rate - Oxygen - Skin temperature/ perspiration - Core temperature - Blood glucose - Mobility/activity quality - Mobility/activity quantity - Symptoms (specify) - Location (in the home) - Location (outside the home) - Diet quantity - Diet quality - Weight - Body composition - Sleep quantity - Sleep quality - Ambient temperature - Ambient light - Ambient noise - Other ambient - Social engagement quantity - Social engagement quality - Medication adherence - Other (describe) | - Individual only - Individual + other (e.g. Caregiver) - Other only (e.g. Caregiver) - Home/external entity - Other (describe) | - Watch wearable - Other wearables - Smartphone - Tablet - Ambient/ environmental sensors - Glucometer - Wearable vital signs devices - Other (describe) | - Self-monitoring individual - Self-monitoring individual + other/caregiver - Caregiver - Healthcare provider**‡** - Allied health professional**§** - Research team - Rural hospital/clinic - Community hospital/clinic - Academic hospital/clinic - Unspecified hospital |
| **Geographic location of target end-user? Is rural defined using operational definition?** | **At what geographic level is rural defined?*†** | **What definition is used to classify rural?*** | **Was stakeholder input obtained? (Associated DOI)** | **If yes, when was stakeholder input obtained?*** | **What type of stakeholder input was provided?*** | **Do they reference any of the following frameworks/ theories?*** |
| - Write-in - Yes - No - Unclear | - County - Census tract - Zip code - Regional (must be defined region with recognition as rural area (e.g. Appalachia) | - Rural-urban commuting areas (RUCA, USDA) - FORHP definition (HRSA) - Core-based statistical areas (OMB) - CDC rural classification scheme - Rural continuum codes (RUCC, USDA) - Urban areas and urban clusters (census bureau) - Frontier and remote access codes (USDA) - USDA unspecified - Unclear | - Yes - No | - Planning - Active - Deployment - Unclear | - Patients   - Were patients rural? (Yes/ No/Unclear) - Informal Caregivers   - Were the informal caregivers rural? (Yes/ No/Unclear) - Healthcare Clinicians   - Were the clinicians rural? (Yes/No/ Unclear) - Community Members   - Were the community members rural? (Yes/No/ Unclear) - Unclear | - Human-centered design - User-centered design - Design thinking - Community-based participatory research - No/unclear |
| **Did they consider or adapt for broadband access?** | **What broadband considerations or adaptations were made? † Did they exclude participants who did not have broadband?** | **Did they discuss financial considerations of RHMS (e.g. insurance coverage)? If yes, what did they discuss and/or do?** | **Did they adapt for other rural cultural/ contextual considerations?*** | **Lessons learned: barriers to RHMS identified†** | **Lesson learned: facilitators to RHMS identified†** | **Relevant Study Limitations and Recommended Next Steps (as identified by author)** |
| - Design phase; discussed - Design phase; not discussed - Discussed and adaptions made - Discussed but no adaptions made - Not discussed or adapted - Not discussed or adapted   Not necessary for system functioning | - Write-in - Yes - No - All participants had broadband - Design phase; not yet relevant | - Yes - No - Unnecessary for system operation | - Distance to care - Community distrust in healthcare - Privacy/ security concerns - Digital literacy - No/unclear - Other (describe) | - Write-in | - Write-in | - Write-in |

*Select all that apply
†Per study author description
**‡** Healthcare provider: An individual licensed to deliver healthcare services, such as diagnosis, treatment, and preventive care, including physicians, physician assistants, nurse practitioners, and nurses.^58^
**§** Allied health professional: diverse group of non-physician, non-nurse healthcare workers who promote health and wellness, and support healthcare systems across various settings.^59^
